# The Impact of a Mobile Shading System and a Phase-Change Heat Store on the Thermal Functioning of a Transparent Building Partition

**DOI:** 10.3390/ma14102512

**Published:** 2021-05-12

**Authors:** Michał Musiał, Lech Lichołai

**Affiliations:** The Faculty of Civil and Environmental Engineering and Architecture, Rzeszow University of Technology, 35-959 Rzeszów, Poland; Lech.Licholai@prz.edu.pl

**Keywords:** construction, solar shutters, solar radiation energy, heat storage, phase change material, building window shading systems

## Abstract

The article presents the results of multi-month field tests and numerical analyses describing the thermal functioning of mobile shading systems for building windows containing a phase-change heat accumulator. The experiments were conducted in the summer period with temperate climate conditions in Rzeszów (Poland). The tested shading system was dedicated to the daily life cycle of residents, taking into account both the need to illuminate the rooms with natural light and reducing the undesirable overheating of the rooms in the summer. The obtained empirical results showed a reduction in room overheating in the summer period by 29.4% from composite windows with a phase-change heat accumulator and a mobile shading system as compared to the reference composite window with an analogous mobile shading system. The database of empirical results allowed for the creation and verification of a numerical model of heat conversion, storage and distribution within the composite window containing phase change material and a mobile shading system. The verified model made it possible to analyse the thermal functioning of the modified transparent partitions in cool temperate, temperate and subtropical climates. The article is a solution to the problem of undesirable overheating of transparent building partitions by efficient storage and distribution of solar radiation energy thanks to the use of a mobile shading system and a phase change material, while presenting a useful tool enabling the prediction of energy gains in different climatic conditions.

## 1. Introduction

The need to reduce the costs of thermal operation of buildings, resulting from increasingly restrictive legal regulations and the increase in prices of supplied energy, makes it necessary to use new energy-saving material and technological solutions in construction. Reductions in the financial, energy and environmental costs of using buildings can be achieved through more efficient use of renewable energies such as solar energy. In this approach, the modification of the structure or functioning system of transparent building partitions may provide great opportunities for the effective use of solar radiation gains as part of passive solutions. Transparent partitions, thanks to their function and structure, transmit the energy of solar radiation, heating up and illuminating the rooms inside the building. For this reason, the skilful use of transparent partitions and accompanying shading elements can contribute to lowering the costs of thermal functioning of buildings in the summer and heating periods. Transparent partitions are elements of thermal casing of buildings, whose temperature and amount of generated heat losses and gains strongly depend on the temporary conditions of the external climate. According to [1,2], the direct gain system is characterised by high instantaneous efficiency and low heat capacity. For this reason, in many types of climates: cool temperate, temperate and subtropical, solutions increasing the thermal inertia of building windows are used. A solution to the problem of reducing the energy demand of buildings, while increasing the comfort of use for their inhabitants, is presented in [3]. The authors, conducting a computational simulation of the results and the analysis using the EnergyPlus™ program, showed that for the Beijing climate, the use of shading systems operating with low-emission thermal insulation glazing for windows can generate energy savings in the range of 30–80%.

Another consideration of the effects of horizontal shading of building windows on the example of the South Korean climate is presented in [4]. The results of experimental tests have shown an increase of the daily period of their shading on a daily basis over the use of using vertical blinds. This translated into energy savings of 13%. The research showed no effect on the energy consumption of using internal window blinds and an increase of 24% in energy consumption in the case of horizontal internal blinds with southern orientation.

The results of research related to the reduction of energy consumption needed to ensure the functioning of buildings through the use of systems covering the building’s windows are presented in [5]. The tests and simulations were carried out for the Indonesian climate, using eQUEST software, which made it possible to conduct multi-criteria analysis of the impact of types of roof structures, glazing and the method of their shading on the energy consumption of residential buildings. The obtained results indicate that in the analysed climate, the greatest impact on the reduction of energy costs of the buildings in question was the appropriate shading of the windows, and then the thermal parameters of the windows of the buildings.

In addition to the thermal aspects, preventing excessive lighting and uncontrolled transmission of solar radiation to the interior of the building is an important problem investigated in numerous scientific works. Reference [6] presents the results of research on shading and anti-glare systems in Australian climatic conditions. The obtained results indicate that the appropriate selection of the shading system during the day increases the comfort of use of the users of the tested rooms, which translates into a reduction of excessive shading of the windows during the day and illumination of rooms with artificial light.

Considerations on various control systems for complex building shading systems located in the United States are presented in [7]. The authors investigated the effect of using various strategies to counteract excessive overheating and over-lighting of offices in six US locations. The presented results prove that the selection of an appropriate shading system control strategy can reduce electricity consumption for cooling the building up to 40%, and the use of external sun screens to 25%.

The influence of phase-change materials (PCMs) on the thermal functioning of transparent building partitions depends, among others, on the physical and chemical properties of the PCM. For this reason, empirical results allowing assessment of the impact of PCMs, differing from each other in only one parameter, on the thermal functioning of the building’s windows are valuable. In [8], an empirical and numerical comparison was made of three PCM MG29 paraffins, differing only in the value of the melting/freezing enthalpy and pure water, applied in the inter-pane space of the window units. The results from the summer and winter periods showed a reduction of heat transfer through the window with PCM compared to water during sunny days by 18.3% and an increase in heat transfer through the window with PCM compared to water during rainy days by 4.9%.

Another method of using window shades, described in [9], are vertical meshes placed between the glass panes of glazing units. As empirically proven, such a solution, operating in China and the USA, can reduce the cooling load of the considered buildings by 37.8% and 24.8%, respectively.

Another solution used to reduce the cost of lighting rooms and improve the comfort of their use, described in [10], is the use of mobile and horizontal reflective blinds reflecting direct solar radiation towards the ceiling.

In turn, in order to increase the efficiency of shading systems for building windows in warm climates, controlled shading systems are used based on the variable arrangement of the blinds depending on the latitude, time of day, year and light demand [11,12]. Paper [13] shows that the use of such a system on the example of the Indian climate can reduce the costs of space cooling by 7.57%. Research on a similar solution, but with fixed external blinds, tested in a cool Chinese climate, were presented in [12]. The obtained results confirmed that the most effective angle of external window blinds was 90°, which increased energy savings by 21.77% in the case of air-conditioned rooms.

Shading systems are an important part of transparent building partitions, which, when used in a skilful way, can contribute to lowering the costs of heating and cooling rooms [14]. More and more modern shading systems for building windows are being implemented in the construction industry, e.g., with phase change materials [15,16,17], electrochromic installation [18], taking into account air flow models [19] and greenery systems [20]. The authors in [20] presented a system of mobile shutters as a modification of the often-used vertical greenery systems. The solution based on the influence of the movement, with greenery systems shading the window, allowed a reduction of the cooling capacity during summer in adjacent rooms of 11.5%.

An important aspect of the assessment of the effectiveness of the modernisation of windows and roller shutters in buildings are the costs of their life cycle. In paper [21] an analysis of empirical and numerical results was carried out in the EnergyPlus™ program on the example of the climate of Brazil. The obtained results proved that wooden windows with an external shading system are a favourable solution in terms of air-conditioning costs and life cycle costs.

Transparent building partitions, due to their function and structure, are building elements that generate high heat losses during the night hours during the heating season, on the other hand, they illuminate the room and generate thermal gains from solar radiation. The aforementioned facts suggest that transparent partitions or elements cooperating with them, modified with phase change materials, may generate greater energy gains than the use of PCM in other building partitions. In [22], a SWOT (strengths, weaknesses, opportunities and threats) analysis of nine different solutions for the application of phase change materials in inter-pane spaces of transparent partitions, with the use of airgel and transparent thermal insulation, was carried out. The presented results included the strengths and weaknesses of the tested solutions, paying attention to the gains associated with increasing the thermal inertia of windows and reservations related to the performance of these solutions in the winter season. Paper [22] also deals with visual aspects and technological problems related to the application of the tested solutions.

Heat storage within PCM phase change enthalpy is used both in construction and other industries. The parameters determining suitability are both the value of the enthalpy of the phase change and the temperature range in which it occurs. In the case of building elements, depending on the type of climate and the place of PCM application, the range is between 16–28 °C [15,16,23,24,25,26,27,28,29]. The use of organic phase change materials to modify building partitions is associated with the need to ensure the necessary heat flow between PCM and the external environment [27]. The researchers in [30] solved the above problem by using aluminium honeycomb panels, which allowed partial avoidance of the problem of not using the full potential of heat absorption, storage and reception in the PCM structure. One of the possibilities of reducing the costs of air-conditioning and heating rooms in buildings is the use of PCM in the structure of window blinds. In [17] an analysis of the functioning of this solution both in winter and summer was presented on the example of the climate of South Korea. The researchers demonstrated a cooling load reduction of 44%, thereby increasing the period of thermal comfort by 34%. Research on the solution of a ventilated window with a phase change material on the example of the Copenhagen (Denmark) climate is presented in [31]. Researchers analysed a constant amount of PCM with a heat capacity of 3.19MJ in plate heat exchangers of thicknesses of 5, 10 and 20 mm. The presented results confirm the most favourable energy effect when using the thinnest accumulation plates.

Especially for locations with a warm climate, transparent partitions are components of external walls that can overheat the interior of the building. Paper [32] presents a computational procedure for the functioning of a liquid flow system within the inter-pane space of a 9 m^2^ window unit. The heat obtained and cold from a low-temperature source and an active solar energy system allowed the window temperature to stabilise on a daily basis. The results of the analysis proved the achievement of zero heat gain in the tested room. A similar solution of flow cooling of transparent partitions was described in [33], and the obtained results prove that the amount of heat flowing through the examined window in summer was reduced by approx. 43%. In turn, [34] presents the results of research on the solution of building windows, modified with silica airgel and phase change material. During the research, the effectiveness of PCM airgel packets was demonstrated only in cold climates.

Modelling the thermal functioning of buildings and the installation systems contained in them is possible thanks to advanced software systems such as IDA ICE, described in [27], EnergyPlus™ [7,11,13,19,35,36] and MatLab [25], enabling the analysis of the functioning of the building and its individual parts and partitions according to available climatic data before and after PCM modification. The results of modelling the heat demand of a building on the basis of meteorological data using the Wiener process are described in [37]. The presented system included window shading devices, a heat store and a ventilation system. The verification of the model 90% confirmed the theoretical cost savings related to taking into account the described forecasts.

A strategy for the efficiency and control of a PCM ventilated window system developed in [37] with the use of EnergyPlus™ software has demonstrated an increase in the energy efficiency of a building’s windows. The use of an hourly airflow control system allowed for energy savings of 62.3% for the summer period and 9.4% for the winter period, compared to conventional solutions. An important aspect of the use of PCM in windows is the characteristics of the glazing units and PCM in the context of photothermal conversion of solar energy. The numerical model proposed in [38], based on the equations of the finite difference method, takes into account the influence of the above properties on the PCM phase transformation phenomenon. Comparison of the simulation results of the four-band spectrum model and the conventional grey body model proved that the simulation results of the proposed model were more precisely matched to the empirical results by 9.2%.

Additionally, in order to be able to more accurately reflect the influence of shading systems, with both active and passive windows of buildings, on the thermal functioning of buildings, dynamic models described in [39] are used. They allow for more accurate consideration of modern, innovative window shading systems for the assessment of the energy performance of the building.

To sum up, the cited results of empirical research as well as theoretical and numerical analyses confirm the beneficial effect of using blinds and roller shutters in building windows in the transitional and summer seasons on the example of various climatic zones. Taking into account the possibility of storing the absorbed heat in the structure of blinds and glazing units of building windows using phase change materials and their impact on ensuring thermal comfort, the need to experimentally determine the impact of using a dynamic external shading system for building windows with heat storage systems was noted, and this was carried out in this article. The scope of the research included the assessment of the cooperation of the external shading roller shutter cooperating in parallel with the composite window with the internal PCM heat accumulator, to reduce the cooling demand of rooms and to more accurately match the gains from solar radiation to the profile of demand for them in the building’s rooms.

## 2. Materials and Methods

### 2.1. Materials

- Proprietary phase change material, in the form of a eutectic mixture of propyl palmitate and butyl stearate [25],
- a rectangular container made of 2 mm thick aluminium sheet, dimensions 650 mm × 100 mm × 50 mm, covered with a black matt paint coating,
- an aqueous dispersion of polymer Osakryl OB, manufactured by Synthos (Oświęcim, Poland) [40,41]
- triple-glazed units of the termoflottype: Ug = 0.5 W/m^2^·K, containing a low-emission coating of the structure /4/16Ar/4/16Ar/4/,
- double-glazed units of the termoflottype: Ug = 0.7W/m^2^·K, with a low-emission coating, of the structure /4/16Ar/4/.

### 2.2. Apparatus

- Thermocouple LT 019008 (Ahlborn, Holzkirchen, Germany), (measurement error ±0.1 °C),
- Heat flux density sensor: type FQA020 C (Ahlborn, Holzkirchen, Germany), (measuring error ±2%),
- Almemo 2890-9 recorder (Ahlborn, Holzkirchen, Germany),
- Almemo FLA 613 GS pyarometer (Ahlborn, Holzkirchen, Germany), (measurement error ±5%),
- Thermoregulator Esco (Ahlborn, Holzkirchen, Germany), 4-channel with a hysteresis of 1 °C.

### 2.3. Research Method

An empirical and numerical experimental method was used to obtain information on the impact of mobile shading systems on the thermal functioning of a window with a phase change heat accumulator. The empirical method provided information on the functioning of the tested solution in field conditions, demonstrating its advantages and disadvantages, and also enabled the creation of a database for the construction and verification of the numerical model. In turn, the numerical method made it possible to create, validate and verify the obtained model, thanks to which it will be possible to conduct analyses of the thermal functioning of the considered solution in different climatic conditions, using the data of a typical metrological year.

Daily, cyclical changes in climatic conditions and high values of daily total solar radiation in areas with temperate, cool temperate and temperate climate and subtropical climate, force the use of solutions preventing overheating of building rooms. For this purpose, systems are used to shade transparent partitions of buildings, such as: blinds, roller shutters or extended eaves. The type of shading system used and the way it functions depend on:

- nature of the external climate,
- cardinal direction of the transparent barrier,
- light and heat demand profiles of the building.

**Figure 1:**
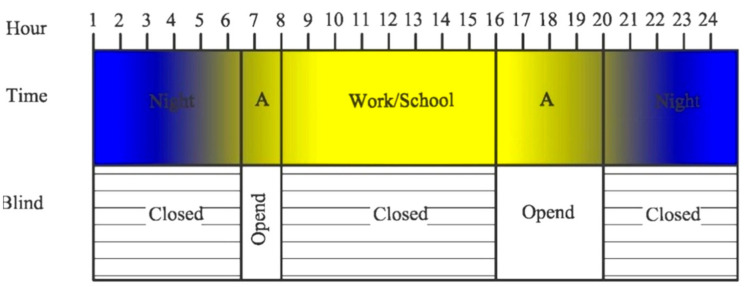
Diagram of the functioning of the external roller shutter during the day; A-Sunlight demand.

The demand for sunlight, depending on the time of day and the lifestyle of the inhabitants, should be taken into account. Consideration of the use of mobile shading systems in windows with a phase change cushion is described in [24,26]. The daily profile of demand for sunlight for people working and studying in a 1-shift, 8-h system was taken into account. The profile refers to the summer period of the considered climatic conditions: (cool temperate, temperate and subtropical). The proposed profile of demand for sunlight assumes opening the external roller shutter between the times 6^30^–8^00^ before the start of work/school activities, then closing it between 8^00^–16^00^, so as not to overheat the rooms during the absence of the household members, then opening the roller blind between 16^00^–20^00^ between the end of work/school and dusk, and then it is closed at night 20^00^–6^30^. A diagram of the daily functioning of the external mobile shading system in the context of the time of day and the daily light demand profile is presented in Figure 1.

Determination of the thermal gains of transparent building partitions modified with phase change materials, related to the use of a shading system, was determined by means of the heat balance in accordance with papers [16,24,26,42]. On the basis of empirical and theoretical data, the heat balance was determined as the integral of the density of the heat flux flowing through the window qt as a function of time, according to Equation (1):

(1) $$Q=A\int_{t=1}^{t=k}q_{t}\;\mathrm{dt}$$

where A—surface, q_t_—heat flux density as a function of time and t—time.

The improvement of the energy efficiency of transparent building partitions containing a phase change material and provided with a shading system, in accordance with [24,26,42], can be determined as the temperature difference between the inside air and the inside of the glazing over time as the number of degree hours S_th_, according to Equation (2):

(2) $$S_{\mathrm{th}}=\overset{t=k}{\underset{t=1}{\sum }}\left|\left(T_{1}-T_{0}\right)\right|$$

#### 2.3.1. Numerical Model

**Figure 2:**
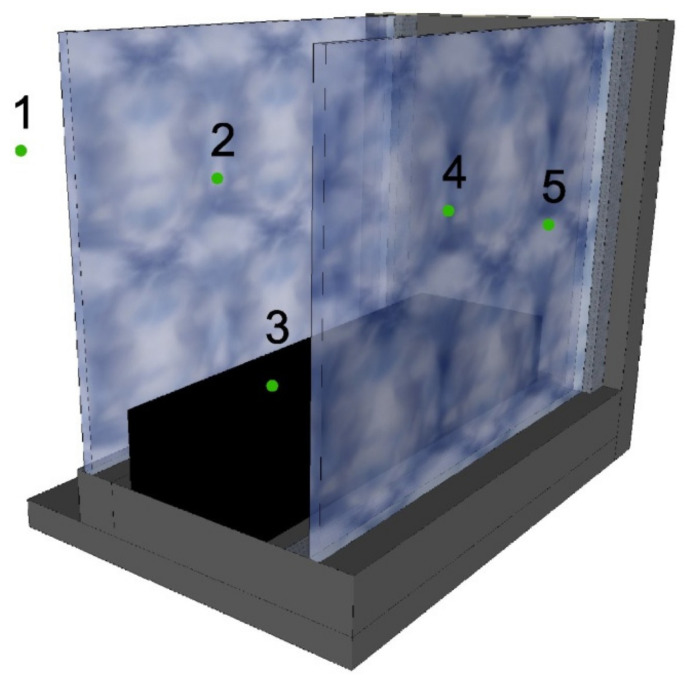
Diagram of the numerical model used; 1 outside air; 2-inner surface of the outer glazing unit; 3-heat accumulator with phase change material; 4- inner surface of the internal glazing unit; 5-indoor air.

The numerical model of the thermal functioning of the solution considered in the work: a mobile window shading system with a PCM heat accumulator was developed in the MatLab environment, using the equations of non-stationary heat flow of the finite difference method in an open system. The choice of the numerical environment and the method of describing thermophysical phenomena was dictated by a large number of scientific works in which it was used [11,13,18,24,43]. In the considered solution of using phase change materials with transparent partitions and shading systems, a complex mechanism of heat exchange between individual elements of the building’s window was taken into account. The temperature distribution in the 1-dimensional discrete system of the composite window with a phase change pillow according to papers [24,26,42,44] was calculated according to the Equations (3) and (4), as shown in Figure 2.

(3) $$T_{i}^{t+1}=\frac{\Delta t}{C_{\mathrm{wi}}\cdot \rho_{i}}\cdot \left(\frac{T_{i-1}^{t}-T_{i}^{t}}{R_{\left(i-1;\;i\right)}}+\frac{T_{i+1}^{t}-T_{i}^{t}}{R_{\left(i;\;i+1\right)}}\right)+T_{i}^{t}.$$

(4) $$\Delta t\leq \min\left[\frac{\Delta x_{i}^{2}}{2\;\cdot \frac{\lambda_{i}}{\rho_{i}C_{w,i}}}\right]$$

where R_(i−1; i__)_—heat resistance between points i−1 and i, ϱ_i_—density of point i, C_wi_—specific heat of the material at point i, T^t^_i_—t temperature of point i at time t, Δt—time step, Δx^2^_i_—square of the thickness of the element I and λ_i_—thermal conductivity coefficient i.

The calculation model presented in Figure 2 and expressed by Equations (3) and (4) takes into account the heat exchange by convection and radiation, between the external glazing and the heat accumulator (points 2–3) in Figure 2 and between the heat accumulator and the glazing internal (points 3–4) in Figure 2. Convective and radiation heat exchange, in accordance with the works [26,45], were taken into account in the model by substitute heat resistances.

The equivalent heat resistances took into account the phenomenon of free convection according to Jacob’s formulas [26,45], where the convective heat transfer intensity is a function of the Prandtl and Grashof probability numbers. The radiation phenomenon was taken into account in accordance with [26,45] as a function of temperature, which takes into account: black body radiation factor, replacement emissivity, and angle radiation factor (so-called configuration factor).

**Figure 3:**
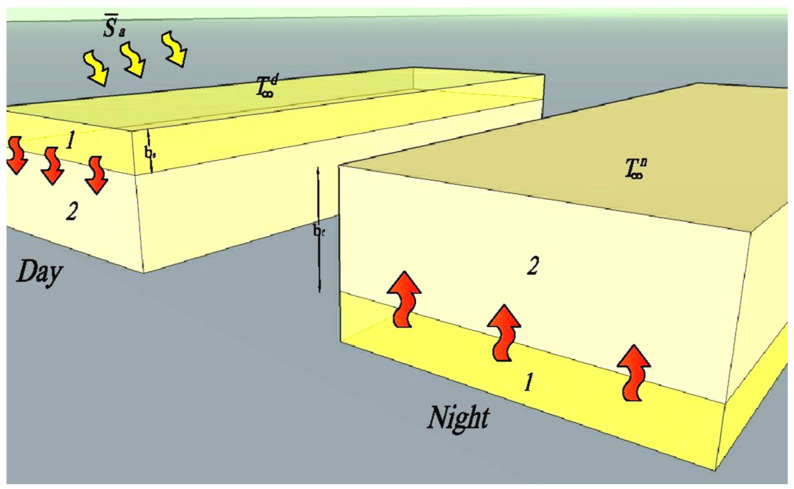
Diagram for determining the boundary of liquid and solid phase PCM; 1-liquid phase; 2-solid phase, according to the concept [2].

One of the methods of modeling heat distribution within phase change materials is the solution to the problem proposed by Stefan cited in [2,26,42,46]. This solution allows the determination of discrete grid points of the PCM system, which for specific time steps create an “x” boundary between the solid and liquid phase of the material. This in turn makes it possible to determine, for individual time steps, the amount of heat stored in the PCM accumulator. The diagram for determining the solution to this problem, according to [2], is shown in Figure 3 and described by Equation (5):

(5) $$\frac{\partial T}{\partial t}=\frac{\lambda_{f}}{C_{f}\varrho_{\mathrm{mzf}}}\frac{\partial^{2}T}{\partial x^{2}}\mathrm{dla}\;\mathrm{for}\;0\;<\;x\;<X.$$

Applying the boundary condition to the PCM layer adjacent to the side surface of the heated accumulator, in accordance with [2], Equation (6) was obtained for the thickness of the liquid PCM layer at time t during charging of the heat accumulator:

(6) $$\frac{U_{u}}{2\lambda_{f}}{\;b}_{f}^{2}+{\;b}_{f}=\frac{t_{d}}{\varrho_{\mathrm{mzf}}\Delta H}\left[{\;\bar{S}}_{a}-U_{u}\left(T_{\mathrm{pf}}-T_{\infty }^{d}\right)\right]$$

In turn, when determining the thickness of the solidified PCM layer, at time t, while the heat accumulator was cooling down, it was determined according to [2] with Formula (7):

(7) $$\frac{U_{u}}{2\lambda_{f}}{\;b}_{s}^{2}+{\;b}_{s}=\frac{t_{n}\cdot U_{u}}{\varrho_{\mathrm{mzf}}\Delta H}\left(T_{\mathrm{pf}}-{\;T}_{\infty }^{n}\right)$$

where ϱ_mzf_—PCM density, λ_f_—PCM heat transfer rate, C_f_—thermal capacity of the PCM, T_pf_—melting/freezing points of the PCM, ${\;\bar{S}}_{a}$—additional heat flux, b_s_—thickness of the solidified PCM layer, b_f_—thickness of the melted PCM layer and U_u_—storage unit heat loss factor.

The influence of the thermal operation of the heat accumulator with a phase-change material on the heat distribution of the composite window was taken into account in Equation (3) according to Figure 3 by introducing in Equation (3) against point 3 instead of specific heat, the bondedtemperaturefunction C_wi.PCM_, Equation (8).

**Figure 4:**
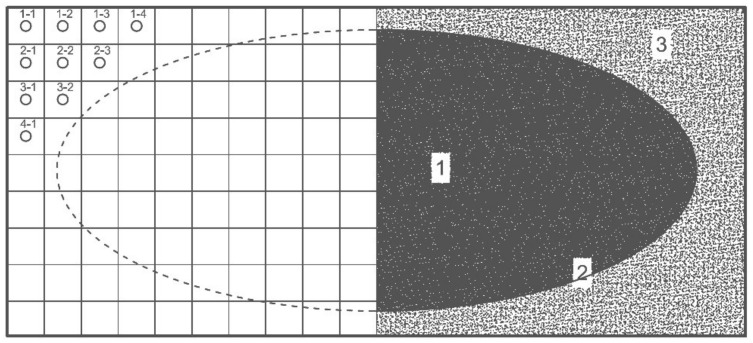
On the left side: Diagram of a discrete grid of a heat accumulator with a phase change material; On the right side there is a diagram of the changes in the physical state of the PCM, in the cross section of the heat accumulator; 1-solid phase change material; 2-concentration border states; 3-liquid phase change material.

Due to the imprecise nature of the phase change of the proprietary phase change material in the heat accumulator [24], it was necessary to determine the time-varying area of its phase change, taking into account the complex heat exchange within individual points of the 2-dimensional discrete grid of the heat accumulator, according to the diagram in Figure 4

The phenomena of complex heat exchange within the inter-pane space and anaccumulator with a phase change material, which takes into account the heat transfer by conduction, convection and radiation, according to the work of Pogorzelski and Jacob [46], were presented in [26]. In the tested solution of using phase change materials in cooperation with transparent barriers, the thermal functioning of phase change materials was expressed according to the Equations (8)–(12) described in [2,26,42]:

(8) $$T_{i,j}^{t+1}=\frac{\Delta t}{C_{\mathrm{wi},j}\cdot \rho_{i,j}}\cdot \left(\frac{T_{i,j-1}^{t}-T_{i,j}^{t}}{R_{\left(i,j-1;\;i,j\right)}}+\frac{T_{i,j+1}^{t}-T_{i,j}^{t}}{R_{\left(i,j;\;i,j+1\right)}}+\frac{T_{j,i-1}^{t}-T_{j,i}^{t}}{R_{\left(j,i-1;\;j,i\right)}}+\frac{T_{j,i+1}^{t}-T_{j,i}^{t}}{R_{\left(j,i;\;j,i+1\right)}}\right)+T_{i,j}^{t}$$

(9) $$\Delta t\leq \min\left[\frac{\Delta x_{i,j}^{2}}{2\;\cdot \frac{\lambda_{i,j}}{\rho_{i,j}C_{w,i,j}}}\right]$$

(10) $$C_{\mathrm{wi}.\mathrm{PCM}}=\left\{\begin{array}{ccc} m_{s}\cdot C_{W.S}\cdot \left(T_{T}-T_{0}\right) & \mathrm{if} & T_{\mathrm{PCM}}>T_{T}\\ m_{T}\cdot \Delta H_{T} & \mathrm{if} & T_{\mathrm{PCM}}=T_{T}\\ m_{l}\cdot C_{W.l}\cdot \left(T_{l\;}-T_{T}\right) & \mathrm{if} & T_{\mathrm{PCM}}<T_{T} \end{array}\right.$$

where T_T_—phase change temperature of the PCM, C_wi,PCM_—specific heat of the PCM and ΔH_T_—PCM phase change enthalpy:

(11) $$\Delta H_{T}=H_{i}^{t+1}-H_{i}^{t}$$

(12) $$\Delta H_{T}^{t+1}=\left\{\begin{array}{cc} \overset{T^{t+1}}{\underset{T=0}{\int }}h{\left(T\right)}_{1}\mathrm{dT}-\overset{T^{t}}{\underset{T=0}{\int }}h{\left(T\right)}_{1}\;\mathrm{dT} & \left|\begin{array}{c} h{\left(T\right)}_{1\;}\to \;\mathrm{for}\;\mathrm{melting}\\ {\mathrm{when}\;T}^{t+1}>T^{t} \end{array}\right.\\ \overset{T^{t+1}}{\underset{T=0}{\int }}h{\left(T\right)}_{2}\mathrm{dT}-\overset{T^{t}}{\underset{T=0}{\int }}h{\left(T\right)}_{2}\;\mathrm{dT} & \left|\begin{array}{c} h{\left(T\right)}_{2}\to \;\mathrm{for}\;\mathrm{clotting}\\ {\mathrm{when}\;T}^{t+1}\leq T^{t} \end{array}\right. \end{array}\right\}$$

Finally, knowing the temperature maps of the discrete mesh, the cross-section of the heat accumulator with the PCM, it was possible to link these heat distribution phenomena of the 2D model with the one-dimensional model of heat transfer within the composite window.

In the case of the tested solution, the functioning of the shading system on a daily basis was described using the conditional time function, in accordance with Equation (13):

(13) $$F_{\mathrm{BLIND}}\left(t\right)=\left\{\begin{array}{l} \begin{array}{ll} \mathrm{OPENED} & \begin{array}{ll} \mathrm{if} & t \end{array} \end{array}\in \left(6.30-8.00;16.00-20.00\right)\\ \begin{array}{lll} \mathrm{CLOSED} & \mathrm{if} & t \end{array}\in \left(8.00-16.00;20.00-6.30\right) \end{array}\right.$$

Then, the verification of the obtained model and the scope of its application was carried out using the analysis of the fit of empirical and theoretical values (obtained from the model) using the quasi-Newton method described in [26]. Within the scope of the fit analysis, the minimum error function was determined in the form of the sum of squared differences between the experimentally measured values T_E_(t) and obtained from the numerical model T_N_(t), according to Equations (14) and (15):

(14) $$\min y=\overset{n}{\underset{t=1}{\sum }}{\left[T_{E}\left(t\right)-T_{N}\left(t\right)\right]}^{2}$$

(15) $$R^{2}=1-\frac{\sum_{t=1}^{n}{\left({\;\hat{y}}_{t}-\;\bar{y}\right)}^{2}}{\sum_{t=1}^{n}{\left(y_{t}-\;\bar{y}\right)}^{2}}$$

where ${\;\hat{y}}_{t}$—theoretical value of the explanatory variable, y_t_—actual value of variable y at time t and y—arithmetic mean of the value of the independent variable.

#### 2.3.2. Field Tests

**Figure 5:**
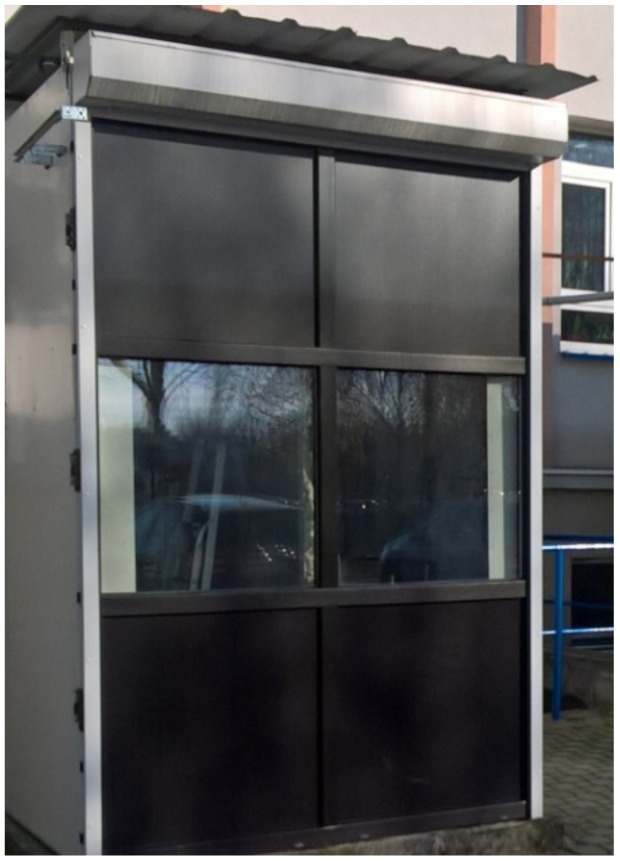
Photograph of the field test chamber.

The research was conducted in the transitional and summer periods (April–October 2019) in a large-scale test chamber located in Rzeszów (Poland). The chamber is made of a steel frame structure, thermally insulated with a 20 cm layer of mineral wool. The chamber on the south side has glazing in the form of two identical composite windows. In each of the two windows, triple-pane glazing was installed as external glazing, and double-glazed internal units. During the tests, one of the windows contained a phase-change heat accumulator and the other served as the reference window. Identical PVC windows are used in both windows. An electronically controlled external roller shutter was installed on the south side of the test chamber. A photograph of the test chamber is shown in Figure 5.

**Figure 6:**
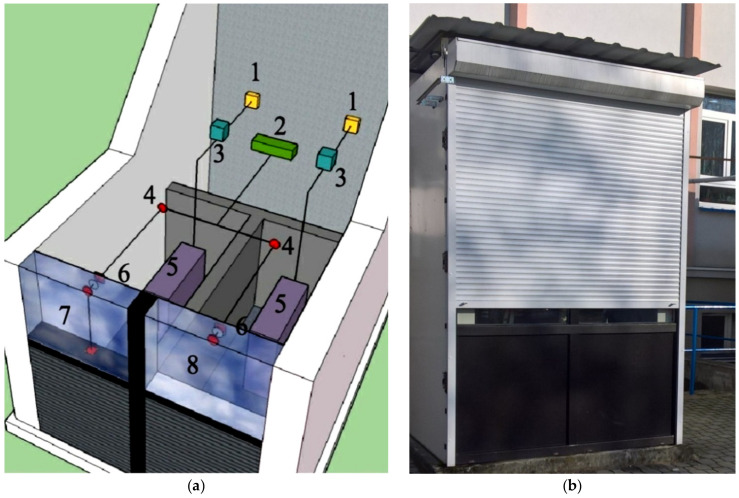
(**a**) Diagram of a field test chamber with an internal PCM heat accumulator; 1-energy consumption recorder; 2-measurement data recorder; 3-thermoregulator; 4-temperature sensor; 5-oil heater; 6-temperature and heat flux density sensor; 7-window with PCM heat accumulator; 8-reference window; (**b**) Photograph of the external roller shutter, test chamber.

In order to conduct meaningful and reliable field tests of the thermal functioning of the composite window solution with a phase change cushion, together with a mobile shading system, the interior of the test chamber was divided into two identical cubic volumes, so that each had one window. The two spaces were separated by a partition made of a 15 cm layer of extruded polystyrene. During the tests, the following values were recorded in 10-min time steps: the intensity of solar radiation falling on the plane of the windows, the temperature values of: external air, internal air, internal glazing units, PCM accumulators and air in the spaces between the glass panes. In addition, the values of heat flux density on the internal surfaces of the internal glazing units, reference windows and with a PCM accumulator were recorded. A diagram of the arrangement of the sensors, recorder and additional equipment is presented in Figure 6.

## 3. Results

### 3.1. Empirical Results

**Figure 7:**
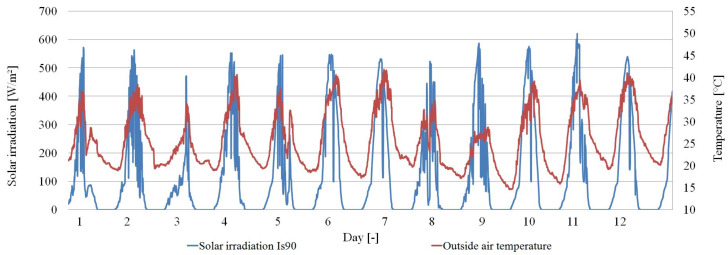
Graph of the intensity of solar radiation on the S90 surfaces and the temperature of the outside air.

**Figure 8:**
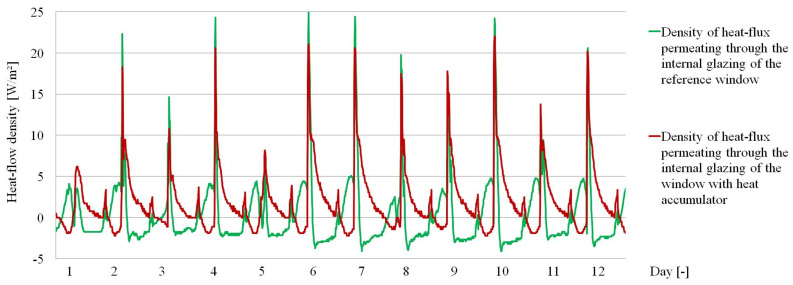
Values of the heat flux density recorded on the internal glazing of the window with the heat accumulator and the reference window.

**Figure 9:**
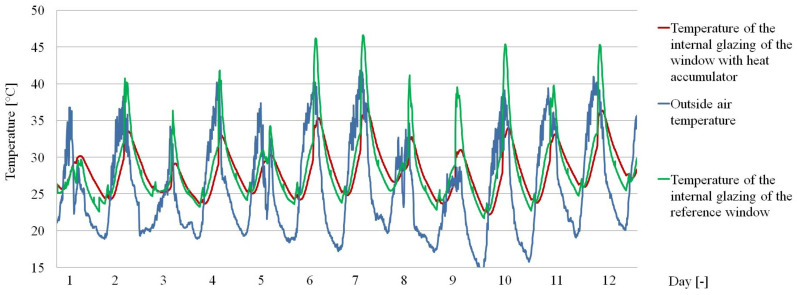
Temperature values recorded on the internal glazing of the window with the heat accumulator and the reference window.

The obtained empirical results are presented in a graphical and tabular form. Figure 7, Figure 8 and Figure 9 show the thermal functioning of the tested partitions in the transitional and summer periods.

The results presented in the graph in Figure 8 show changes in the density of fluxes from the penetrating heat. A reduction of the peaks during daylight access was observed along with a “flattening” of the heat flux density curves per unit time in the window with the PCM accumulator compared to the reference window. This fact caused a significant reduction in the time in which the heat flux passing through the PCM window changes its direction, compared to the reference window. The confirmation of the results presented in Figure 8 are the values of temperatures recorded in the same time, presented in Figure 9.

### 3.2. Results of Mathematical Analysis

**Figure 10:**
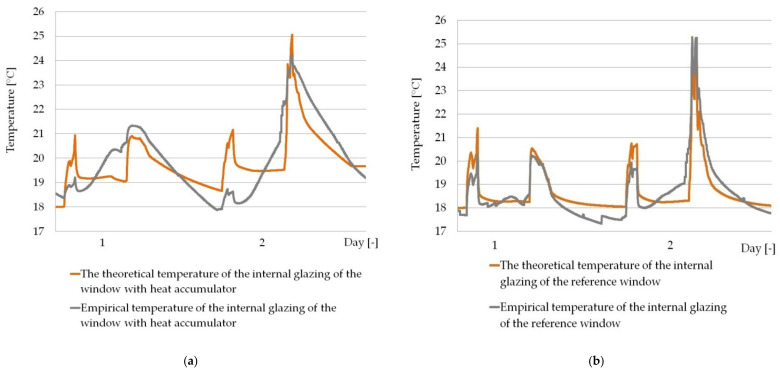
(**a**) Graphs of the measured and modelled temperature of the internal glazing surface of the window with the PCM heat accumulator; (**b**) Graphs of the measured and modelled temperature of the internal glazing surface of the reference window.

**Figure 11:**
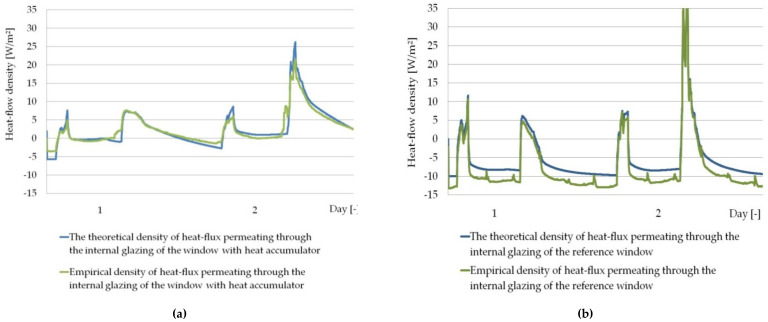
(**a**) Graphs of the measured and modelled heat flux density at the internal glazing surface of the window with the PCM heat accumulator; (**b**) Graphs of the measured and modelled heat flux density at the internal glazing surface of the reference window.

Using the validated and verified numerical model described in [26], functioning within the desired significance level p=5%, in the case of composite windows with a PCM accumulator, the authors checked the possibility of its adaptation to predict the thermal response of the modified PCM window operating in the daily shading system cited in point Section 2.3.1. Figure 10 and Figure 11 show a summary of heat flux density and temperature graphs, empirical values and values obtained from the model, for randomly selected two-day periods of time.

**Figure 12:**
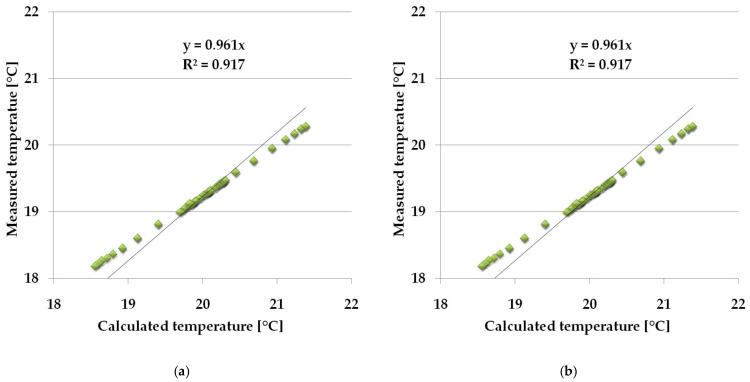
(**a**) Graph of the fit of empirical and theoretical values of the temperature of the window with a PCM accumulator; (**b**) Graph of the fit of empirical and theoretical values of the reference window temperature.

The results of the fit of empirical and numerical values, temperature and heat flux density presented in Figure 10 and Figure 11 for randomly selected days prove that the empirical and theoretical values are well matched. Additionally, in order to parametrically define the measure of the fit of the empirical values to the theoretical ones obtained from the model, an analysis of their fit was carried out using the quasi-Newton method in order to find the minimum error function, according to the procedure presented in Section 2.3.1. The results of the analysis, along with the obtained function formulas and the values of the determination coefficients, are presented in Figure 12.

The presented results of the directional coefficients of the error function and the determination coefficient close to unity indicate a good fit of the empirical and theoretical values obtained from the numerical model.

### 3.3. Assessment of the Impact of PCM Shading Systems in Various Climatic Conditions

**Figure 13:**
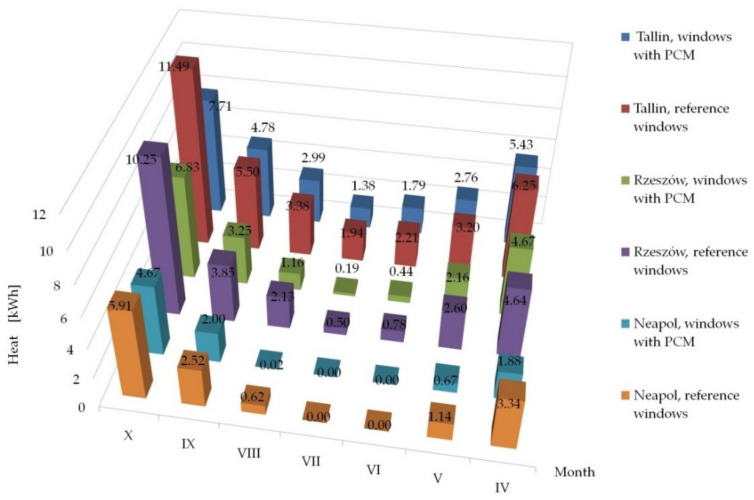
Summary of monthly heat losses (the absolute value) through windows with a PCM accumulator and reference windows in Tallinn, Rzeszów and Naples.

**Figure 14:**
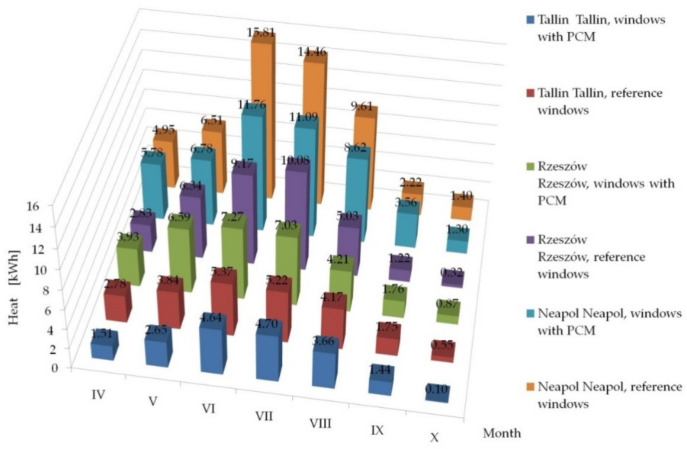
Summary of monthly heat gains through windows with a PCM accumulator and reference windows in Tallinn, Rzeszów and Naples.

**Figure 15:**
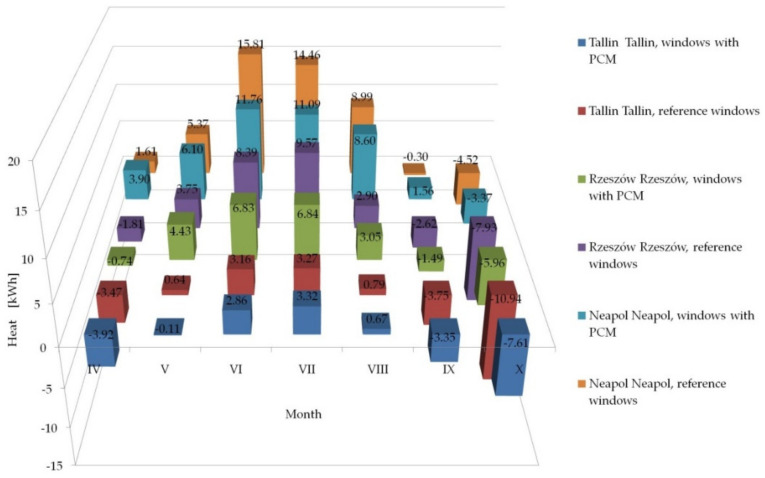
Summary of monthly heat balances through windows with a PCM accumulator and reference windows in Tallinn, Rzeszów and Naples.

Having knowledge of the adequacy of the considered numerical model, in accordance with paper [26] and checking the possibility of its application in the case of external shading systems, operating in the summer and transitional seasons, by checking the fit of empirical theoretical values with the quasi-Newton method in Section 3.2, we proceeded tosimulate the impact of the tested solution in other types of climates. The impact was analysed of the use of windows with a heat accumulator made of PCM and a reference window equipped with mobile shading systems on the gain, loss and heat balance of the modified windows, operating in the following climates: cool temperate, temperate and subtropical climates. The analyses were conducted on the basis of hourly data of a typical metrological year for the locations of Tallinn, Rzeszów and Naples from [47]. The results of the conducted analyses are presented in Figure 13, Figure 14 and Figure 15 and Table 1, Table 2 and Table 3.

The presented results confirm the stabilising changes in the heat balance of transparent partitions and the effect of the phase change material compared to the reference partitions on a monthly basis. Moreover, for the considered locations of temperate and subtropical climates, a decrease in the value of monthly heat balances of PCM windows was noted in relation to the reference windows during the heating season. A smaller impact of the ability to limit overheating of windows with PCM and shading systems was noticed compared to reference windows with a shading system in the summer period for Tallinn than for Rzeszów and Naples, which was due to lower gains from solar radiation and lower demand for space cooling in Tallinn than in the other two locations.

## 4. Discussion

The obtained results of long-term empirical research carried out in a large-scale test chamber proved the correctness of the thesis about the minimising intensity of heat exchange as the effect of a window with a PCM accumulator compared to a reference window. The result of this fact is a more favourable adjustment of solar radiation gains to the building’s demand profile and, consequently, reduction of costs of cooling and air-conditioning of the building in the summer period.

The developed model of non-stationary, complex heat transfer through windows with a heat accumulator containing PCM with a wide range of melting and freezing points cooperating with a mobile shading system is reliable. This was proved during the analysis of the fit of empirical and theoretical values with the quasi-Newton method.

An additional utilitarian aspect of the work is to simulate the effect of windows combined with PCM accumulators and a mobile shading system on their heat balance, on the example of cool temperate, temperate and subtropical climates.

The results of the analysis of the three considered locations showed a reduction in heat losses in some of the PCM windows with a mobile shading system compared to the reference window with a mobile shading system, in all the considered types of climates. Additionally, in the location of Rzeszów and Naples, an increase in heat gains generated by the PCM window was recorded compared to the reference window during the transitional months of the summer and heating periods (April, May, September, October). The opposite situation was observed in the summer period on the example of the months: (June, July, August). On the other hand, the example of Tallinn (cool temperate climate) showed a reduction in heat gains generated by the PCM window compared to the reference window, during both the transitional and the summer periods.

## 5. Conclusions

Long-term experimental studies of the operation of a composite window with a PCM heat accumulator and an external shading system have proven the energy benefits of this solution. The calculation model, verified on the basis of separated empirical data, allowed to determine the differences in the heat balances of the tested windows with PCM and the reference windows.

Summing up, in each of the considered locations there was a decrease in the amount of heat flowing inside the building through the PCM window, compared to the reference window, in the entire analysed summer period (from June to August), respectively for Tallinn 5.5%, for Rzeszów29,4% and for Naples 24.8%. The discrepancies in the values of heat balance differences between PCM windows and reference windows in individual climate types result from the thermo-physical properties of the proprietary PCM (melting/freezing enthalpy value and the temperature range in which the phase change occurs) and the combination of glazing units used.

The presented empirical and theoretical results, as well as the conducted analyses, in addition to their cognitive values, allowed the development of a tool that can be used in the design of new buildings and thermo-modernisation of existing ones.

## 6. Patents

Musiał M. Phase-change material and method of producing phase-change material nr: P.425190 from 12 April 2018.

## Figures and Tables

**Table 1:** Heat losses through windows with a PCM accumulator and reference windows operating in cool temperate (Tallinn), temperate (Rzeszów) and subtropical (Naples) climates.

Month	Tallinn		Rzeszów		Naples	
	Window with PCM [kWh]	Reference Window [kWh]	Window with PCM [kWh]	Reference Window [kWh]	Window with PCM [kWh]	Reference Window [kWh]
IV	−5.43	−6.25	−4.67	−4.64	−1.88	−3.34
V	−2.76	−3.20	−2.16	−2.60	−0.67	−1.14
VI	−1.79	−2.21	−0.44	−0.78	0	0
VII	−1.38	−1.94	−0.19	−0.50	0	0
VIII	−2.99	−3.38	−1.16	−2.13	−0.02	−0.62
IX	−4.78	−5.50	−3.25	−3.85	−2.00	−2.52
X	−7.71	−11.45	−6.83	−8.25	−4.67	−5.91
Sum	−26.85	−33.97	−18.67	−22.74	−7.36	−13.52

**Table 2:** Heat gains through windows with a PCM accumulator and reference windows operating in cool temperate (Tallinn), temperate (Rzeszów) and subtropical (Naples) climates.

Month	Tallinn		Rzeszów		Naples	
	Window with PCM [kWh]	Reference Window [kWh]	Window with PCM [kWh]	Reference Window [kWh]	Window with PCM [kWh]	Reference Window [kWh]
IV	1.51	2.78	3.93	2.83	5.78	4.95
V	2.65	3.84	6.59	6.34	6.76	6.51
VI	4.64	5.37	7.27	9.17	11.76	15.81
VII	4.70	5.22	7.03	10.08	11.09	14.46
VIII	3.66	4.17	4.21	5.03	8.62	9.61
IX	1.44	1.75	1.76	1.22	3.56	2.22
X	0.10	0.55	0.87	0.32	1.30	1.40
Sum	18.71	23.68	31.65	34.99	48.89	54.96

**Table 3:** Heat balances through windows with a PCM accumulator and reference windows operating in cool temperate (Tallinn), temperate (Rzeszów) and subtropical (Naples) climates.

Month	Tallinn		Rzeszów		Naples	
	Window with PCM [kWh]	Reference Window [kWh]	Window with PCM [kWh]	Reference Window [kWh]	Window with PCM [kWh]	Reference Window [kWh]
IV	−3.92	−3.47	−0.74	−1.81	3.90	1.61
V	−0.11	0.64	4.43	3.75	6.10	5.37
VI	2.86	3.16	6.83	8.39	11.76	15.81
VII	3.32	3.27	6.84	9.57	11.09	14.46
VIII	0.67	0.79	3.05	3.90	8.60	8.99
IX	−3.35	−3.75	−1.49	−2.63	1.56	−0.30
X	−7.61	−10.94	−5.96	−7.93	−3.37	−4.52
Sum	−8.14	−10.29	12.95	13.26	39.64	41.44

## Data Availability

Data sharing is not applicable to this article.

## Nomenclature

**Symbol**	**Meaning**	**Units**
A	surface	m^2^
bf	thickness of the melted PCM layer	m
bs	thickness of the solidified PCM layer	m
Cf	thermal capacity of the PCM	W/kg·K
Cw	specific heat of the material	W/kg·K
Cwi,PCM	specific heat of the PCM	W/kg·K
ΔHT	PCM phase change enthalpy	J/g
Q	Heat	Wh
qt	heat flux density as a function of time	W/m^2^
R	heat resistance	m^2^·K/W
R2	determination coefficients	[-]
$S_{a}$	additional heat flux	W/m^2^
Sth	number of degree hours	Kh
t	time	h
T	temperature	°C
Tpf	melting/freezing points of the PCM	°C
TT	phase change temperature of the PCM	°C
Δt	time step	h
Uu	storage unit heat loss factor	W/m^2^·K
Δxi	thickness of the element i,	m
y	arithmetic mean of the value of the independent variable	[-]
${\hat{y}}_{t}$	theoretical value of the explanatory variable	[-]
yt	actual value of variable y at time t	[-]
λ	thermal conductivity	W/m·K
λf	PCM heat transfer rate	W/m·K
ϱ	surface density	kg/m^2^
ϱmzf	PCM density	kg/m^3^
